# Analysis of the Impact of Health Beliefs and Resource Factors on Preventive Behaviors against the COVID-19 Pandemic

**DOI:** 10.3390/ijerph17228666

**Published:** 2020-11-22

**Authors:** Sunhee Kim, Seoyong Kim

**Affiliations:** 1 Department of Local Government Administration, Gangneung-Wonju National University, Gangneung-si 25457, Korea; shkim7675@hanmail.net; 2 Department of Public Administration, Ajou University, Suwon 16499, Korea

**Keywords:** COVID-19, protective action, preventive behavior, health belief model, resource theory

## Abstract

The global spread of COVID-19 requires not only national-level responses but also active compliance with individual-level prevention measures. Because COVID-19 is an infectious disease that spreads through human contact, it is impossible to end its spread without individuals’ active cooperation and preventive behavior. This study analyzes the effects of health beliefs and resource factors on behaviors to prevent COVID-19. In particular, it analyzes how resource factors moderate the impact of health beliefs on preventive behavior. A regression analysis showed that gender (female), age, number of elderly people in one’s family, perceived severity, perceived benefit, self-efficacy, poor family health, media exposure, knowledge, personal health status, and social support positively affected preventive actions, whereas perceived susceptibility negatively affected them. In explaining preventive actions, self-efficacy had the greatest explanatory power, followed by gender (female), knowledge, personal health status, perceived severity, and social support. In addition, an analysis of moderating effects shows that resource variables, such as education level, personal health status, and social support, play moderating roles in inducing preventive actions.

## 1. Introduction

In Korea, the first confirmed case of COVID-19 occurred on 4 January 2020, and the number of confirmed COVID-19 cases and deaths worldwide have been increasing exponentially since then. On 3 September 2020, the World Health Organization (WHO) [1] reported that there had been 25,884,895 confirmed cases of COVID-19 globally, including 859,130 deaths. COVID-19 is different from other pandemic infections such as the 2003 Severe Acute Respiratory Syndrome (SARS) and the 2012 Middle East Respiratory Syndrome (MERS) outbreaks [2]. First, organized, systematic, and scientific knowledge about COVID-19 is lacking, which has caused public fear [3]. Second, the COVID-19 pandemic is different in terms of its high spread rate, low immunity levels that create vulnerability, and rate of recovery [4]. In addition, the low cure potential in confirmed COVID-19 cases has been producing public fear. According to Kwok et al. [5], when respondents in Hong Kong were asked about the possibility of being cured of a COVID-19 infection, 16.0% responded that it was possible, whereas 43% said that it was impossible. In addition, when respondents were asked about recovery from COVID-19, 18.0% said that it was possible, 36% said it was impossible, and 46% expressed a neutral opinion.

People’s responses to COVID-19 are very different from their responses to existing large-scale epidemics. According to Leung et al. [6], in the case of the SARS outbreak (29 March to 4 April 2003), 31% of respondents in Hong Kong expressed that they were very or somewhat likely to contract SARS. In the case of the H1N1 pandemic in 2009, according to Seale et al. [7], 52.9% of 627 Australian respondents felt that they had a “very low to low” risk of acquiring H1N1 and 24.5% said it would very seriously or extremely affect their health.

Along with the rapid spread of COVID-19, health-related authorities such as governments rapidly responded to the disease through regulations requiring citizens to take preventive measures to reduce its spread and impact. These actions include wearing a mask, covering one’s mouth with a sleeve when coughing, washing hands, and social distancing. Many citizens complied with the behaviors recommended by their health authorities. By analyzing survey data collected in India, Jose et al. [8] showed that most of the 638 participants surveyed (93.8%) followed the government’s recommendations to combat COVID-19. According to Czeisler et al. [9], 81.8% of American and Australian respondents (*n* = 7773) surveyed from 2 April to 8 April 2020, were complying with the recommended quarantine or stay-at-home policies (range of responses: 75.5–88.25%). Previous studies have shown that the hygiene-related actions suggested to combat COVID-19 can help prevent infectious diseases. Brienen et al. [10] reported that the population-wide use of face masks can substantially help delay an influenza pandemic. Mask use also reduces the extent of replication, possibly even to the levels sufficient to contain an influenza outbreak.

Despite the significant practical policy implications of preventive behaviors in response to COVID-19, few systematic studies based on theoretical models have been conducted thus far [2,5,8].

The purpose of this paper is to identify how health belief factor and resource factor influence individuals’ preventive actions in response to COVID-19. We focus on comparing the health belief model and resource theory. Second, we examine the moderating role of resource factors in the relationship between predictors and preventive actions. 

## 2. Theory and Hypotheses

### 2.1. Preventive Behavior in the Case of a Pandemic 

In the event of a large-scale infectious disease, it is natural for individuals to take preventive actions. In particular, the general public takes the government’s recommended behavior as the standard. According to Jose et al. [8], when asked whether various activities imposed by the government could be considered cues for people to change their behavior, 544 (80%) respondents felt that the government had helped them implement behavioral changes. Public response and compliance with pandemic regulations existed even before COVID-19. When the 2003 SARS outbreak occurred in Hong Kong, people covered their mouths when sneezing or coughing (78.0%), washed their hands afterwards (74.3%), used soap when washing their hands (74.5%), and wore face masks (75.8%) [6]. In the case of the 2009 influenza A/H1N1 pandemic in Hong Kong, 46.65%, 88.75%, and 21.5% of the respondents (*n* = 999) washed their hands more than ten times a day, wore face masks when having an influenza-like illness, and wore face masks regularly in public areas during the pre-community outbreak phase (7 May to 6 June 2009) [11]. In Australia, people expressed that they would isolate themselves (70.2%) and wear a face mask (59.9%) in the event of pandemic influenza [12]. Citizens’ compliance with government guidance depends on their personal and social contexts. Kwok et al. [5] examined the community’s psychological and behavioral responses in Hong Kong by analyzing survey data from 1715 respondents during the early phase of the COVID-19 pandemic. They reported that many people (>77%) adopted personal hygiene and travel-avoidance practices in China and considered them effective (>90%). 

The main research interest on compliance with COVID-19 preventive behaviors is understanding the causes of these behaviors. First, these studies show that the degree of compliant behavior varies across demographic variables. According to Zhong et al. [13], most Chinese people with high socioeconomic status are knowledgeable about COVID-19, hold optimistic attitudes, and follow appropriate practices. Chen and Chen [14] showed that rural residents are less likely to engage in a thoughtful information appraisal process and take appropriate preventive measures. Cvetković et al. [2] showed that the effects of sex, age, and education vary depending on the type of preventive behavior. According to Kwok et al. [5], variables such as gender, residence area, knowledge, and anxiety determine the adoption of self-protective measures. The mean COVID-19 preventive behavior score was higher among women than among men and among urban residents than among rural residents. In a study by Duan et al. [5], women, city dwellers, and those with higher education levels took more actions recommended by the government. 

Many studies have been conducted on both psychological and demographic variables that influence compliance with COVID-19 preventive behaviors. Shahnazi et al. [15] demonstrated that perceived benefits and self-efficacy increase preventive behavior, whereas perceived barriers and fatalistic beliefs decrease it. Lee and You [16] reported that the psychological responses related to behavioral ones significantly impact the public’s level of preparedness for COVID-19. Zhong et al. [13] showed that COVID-19-related knowledge is significantly related to less-negative attitudes and more preventive practices. According to Duan et al. [17], risk perception is significantly associated with the adoption of protective action recommendations. 

However, the limitations of existing studies are that they are not based on theories and lack comparisons between models and theories [2,5,8,11,12,13,14,15,16,17]. In other words, because prior research has simply focused on specific variables, model-centered comparative studies have not been thoroughly conducted. Therefore, this study adopts the health belief model and resource theory as theoretical frameworks to explain preventive behavior in response to COVID-19.

### 2.2. Health Belief Model and Resource Theory

The health belief model (HBM) is a theoretical model created to explain health-related behaviors, and it focuses on the role of social and psychological attributes in determining these behaviors [18,19]. Rosenstock, Hochbaum, Kegeles, and Leventhal, social psychologists at the U.S. Public Health Service, developed the HBM. Their original goal with the model was to focus on the efforts of people trying to improve public health by understanding why they failed to adopt preventive health measures [20]. In the HBM, people’s beliefs, such as the perceived benefits of health behaviors, obstacles to practice, and self-efficacy, influence their commitment to health-promoting behaviors. The HBM theoretically emphasizes cognitive elements. From a cognitive point of view, people’s behavior often depends on their rational expectations. Empirical studies [21,22,23,24,25] based on HBMs have analyzed changes in health behavior during the SARS and H1N1 pandemics. These studies report that when people are convinced of the severity of the epidemic, they perceive that they are highly susceptible to it, are confident that a protective behavior is effective, and perceive low costs to adopting the precautionary behavior, they are more willing to adopt the recommended behavior [21,22,23,24,25].

In contrast, resource theory emphasizes the assets of individuals, organizations, communities, and countries, by which individuals can respond to infectious diseases. Individuals’ resources include not only their networks and support, material wealth, and physical environments but also their knowledge and social capital, which are more invisible assets. In this study, resource theory is not based on a specific theory in a specific discipline. Our study regards all the theories emphasizing resource factors on judgement as “resource theory.” Therefore, resource theory can include not only micro-level psychological theory, such as that promulgated by Uriel Foa [26]—a social psychological framework for understanding social interactions and the relationships that form between individuals in everyday life—but also macro-level social structure theory, such as social capital by Lin [27]. Risk studies highlight resources as a means of preventing risk. For example, Snyder [28] explained that the lack of resources, such as economic poverty and social inequality, affects people’s relative vulnerability to risk and constrains their opportunities to avoid the hazard. 

The importance of interest in resources and their role is well represented in the research framework on health (in)equality. For example, Solar and Irwin [29] focus on social stratification and socioeconomic status as well as socioeconomic and political contexts, which are macro elements in the determination of health inequality. The social stratification system consists of class, power, prestige, and discrimination, which affect the socioeconomic status such as social class, education, job, and income. The class, education, and income variables presented in this framework correspond to the resources that this study focuses on. Moreover, Kelly [30] proposed that not only social structure related to income and education but also the socioeconomic position, such as gender, ethnicity, and sexuality, and individual social status affect health outcomes and wellbeing. After examining the impact of socioeconomic status on health across the lifespan, Adler [31] refers to parental socioeconomic resources, educational attainment, occupation and income, and retirement income as causes of health inequality. These findings suggest the importance of social resources in health decisions. Those who lack such resources are inevitably susceptible to disease. Vulnerable groups in terms of resources may be exposed to more risk during a pandemic. Enarson and Walsh [32] identify ten groups that are exposed to a higher risk of negative impact during epidemics: older adults, people with limited incomes, children and youth, people with low literacy levels, aboriginal peoples, people with disabilities, women, people with medical dependencies, transient populations, new immigrants, and people who are members of cultural minorities. However, in some cases, irregularities in these resources can also endanger the public. For example, Bavel et al. [33] explain that social networks can amplify the spread of specific behaviors that are both harmful and beneficial during a pandemic. 

As Table 1 shows, the HBM and resource theory are based in different academic fields. The HBM has the advantage of explaining individual behavior in detail as it is based on psychological properties and structures, but it has the disadvantage of overlooking macro-structures that constrain individual thoughts and behaviors. The next limitation is the low predictive power (R^2^ < 0.21 on average) of the existing HBM [34]. Harrison et al. [35] computed mean effect sizes in a meta-analysis of 16 studies, showing that out of 24 mean effect sizes, 22 were found to be positive and statistically significant. However, their effect sizes are usually very small [35]. This low effect size suggests that new explanatory variables are needed. Therefore, LaMorte [36] argued that the HBM did not consider environmental or economic factors that may prohibit or promote the recommended action; HBM determinants are insufficient predictors of behavior [35]. For the most effective use of the model, it should be integrated with other models that account for the environmental context and suggest behavioral determinants. Based on expert judgment, Cummings et al. [37] showed that health behavior can be explained not only by psychological factors, such as perception of symptoms and threat of disease, but also by structural factors, such as social network, knowledge, and demographic characteristics. Another limitation of the HBM is a lack of clear rules of combination and relationship between individual variables [38]. After exploring the relationships/interactions between the HBM variables and their effect size, Orji et al. [34] showed interesting mediating relationships among the HBM’s determinants.

Conversely, resource theory considers the characteristics of the objective social structure in explaining individual behaviors from a more realistic perspective. However, it is limited in that it cannot explain individual behavior in detail. 

### 2.3. Hypotheses from the HBM 

#### 2.3.1. Perceived Susceptibility

Perceived susceptibility refers to one’s subjective assessment of health-related risks [19]. Individuals with high perceived susceptibility are likely to take relevant actions to reduce the risk of contracting illnesses. Conversely, those who view themselves as having a low risk of contraction will have low perceived susceptibility, and they will be passive about engaging in necessary health-related behaviors. For example, after examining the community’s psychological and behavioral responses during the early phase of Hong Kong’s COVID-19 pandemic, Kwok et al. [5] found that perceived susceptibility (89%) and perceived severity (97%) are both high. Prati et al. [39] found that those who assess the risk of pandemic infectious diseases as more probable, more severe, more serious, or having a greater personal impact, are more likely to comply with health-related recommendations.

**Hypothesis 1** **(H1).** *When perceived susceptibility increases, the level of preventive behavior in response to COVID-19 increases.*

#### 2.3.2. Perceived Severity

Perceived severity refers to a subjective assessment of the severity of a health problem and its potential consequences [19]. The more seriously one takes a given health problem, the more one tries to reduce its likelihood of occurrence. Conversely, an individual who underestimates the risk of a disease will engage in less healthy behavior. Perceived severity broadly includes beliefs about the disease itself as well as beliefs about its impact on the work and social roles relevant to the individual [19]. In Prati et al.’s [39] study, perceived severity indirectly affected recommended behavior through people’s affective responses to the H1N1 influenza pandemic in 2009. Duan et al. [15] found that risk perception is significantly associated with the public’s adoption of recommended protective actions. During the 2003 SARS outbreak in Hong Kong, respondents with higher risk perceptions were more likely to take comprehensive precautionary measures against infection [6].

**Hypothesis 2** **(H2).** *As perceived severity increases, preventive behavior in response to COVID-19 increases.*

#### 2.3.3. Perceived Benefits Versus Perceived Barriers

Perceived benefits refer to the evaluative value or sense of efficacy that arises when engaging in health-promoting behaviors to reduce disease risk [18]. According to Jose et al. [8], 36.9% of respondents felt that they were less likely to benefit from health behaviors because no one has a clear understanding of the pathophysiology and epidemiology of COVID-19.

In contrast, perceived barriers refer to an individual’s assessment of obstacles to behavioral change [18]. Perceived barriers include the costs, perceived risk of inconvenience (e.g., side effects of a medical procedure), and discomfort (e.g., pain or emotional upset) related to carrying out a specific behavior. Perceived benefits and perceived barriers have opposite characteristics. If the perceived benefit from an action is greater than the perceived barrier, an actor may engage in behavioral change. Jose et al. [8] showed that 65.9% of respondents expressed perceived barriers; specifically, they identified updates about COVID-19 many times a day through different channels and conflicting information from the COVID-19 “infodemic” as barriers.

**Hypothesis 3** **(H3).** *Greater perceived benefits increase the level of compliant behavior in response to COVID-19, whereas greater perceived barriers decrease the level of preventive behavior.*

#### 2.3.4. Self-Efficacy

Self-efficacy refers to an individual’s perception of his/her ability to successfully carry out a behavior [40]. Confidence in one’s ability to effect outcome changes is a key component of health behavioral change [40]. People who believe that they can control an influenza pandemic are more likely to comply with recommended health-related behaviors. Dryhurst et al. [41] categorized individual and collective efficacy, showing that the former has a positive relationship with COVID-19 risk perception, whereas the latter has a negative relationship. Lau et al. [11] found that greater perceived efficacy of wearing a mask in public places is associated with more frequent mask wearing. Park et al. [22] showed that handwashing frequency increases when its perceived effectiveness increases.

**Hypothesis 4** **(H4).** *As self-efficacy increases, preventive behavior in response to COVID-19 increases.*

#### 2.3.5. Cues to Action

The HBM assumes that the entire motivation process is set in motion by cues to action. Cues to action include a diverse range of triggers for individuals to take action and are often divided into internal (e.g., physical symptoms) and external (e.g., mass media campaigns and advice from others) factors [42]. Moreover, such cues include trusted programs from the authorities, events or information from close contacts, the media, and health care providers [8].

Cues to action related to COVID-19 include direct experiences with COVID-19, media recommendations, and the health status of family members. According to Dryhurst et al. [41], direct experience with COVID-19 influences the relevant risk perception. Media interventions should also be considered because of their small but significant impact on health protection behaviors [28] (p.64). Moreover, cyberchondria, defined as obsessive online searching for health-related information (typically about specific symptoms) and information overload indirectly affects the intention to self-isolate through the aforementioned perceptions [43]. According to Prati et al. [39], exposure to media campaigns negatively affects compliance with recommended behaviors. Finally, family health is one of the most important factors that determine an individual’s behavior. Their study showed that family composition and friends’ concerns influence individuals’ preventive behavioral choices. In addition, worry by family members and friends indirectly affects recommended behaviors.

**Hypothesis 5** **(H5).** *If there are more cues to action, such as knowing someone with a confirmed COVID-19 case, having poor family health status, and having more media exposure, protective actions in response to COVID-19 increase.*

### 2.4. Hypotheses Based on Resource Theory 

#### 2.4.1. Income

Economic wealth serves as a means to effectively respond to large-scale infectious diseases. Economic affordability can reduce external risk by allowing people access to resources to protect against the risks from COVID-19. Chen and Chen [14] showed that respondents with higher incomes report more preventive behaviors, positive attitudes, knowledge, and information appraisal. According to Raifman and Raifman [44], people who live in low-income households are more likely to have an increased risk of illness from COVID-19 relative to those living in higher-income households. In the country of Georgia, the economic situation caused by COVID-19 has led to decreased incomes. Compared with the pre-COVID period, approximately one-fourth of households (23%) had decreased incomes, 65% kept the same income level, and 3% increased their incomes during the pandemic [45]. Such economic poverty undermines the active prevention of COVID-19. However, according to Cvetković et al. [2], income does not have a statistically significant effect on preventive measures.

**Hypothesis 6** **(H6).** *Higher income is positively associated with preventive behavior in response to COVID-19.*

#### 2.4.2. Education

Higher education levels are associated with greater COVID-19 risk and increased awareness of preventive measures. In the case of the 2003 SARS outbreak in Hong Kong, respondents with higher education levels took more comprehensive precautionary measures against infection [6]. This study shows that individuals with an educational background of college or higher have very high probabilities of engaging in preventive behaviors relative to individuals with a university degree or lower. After assessing the perceptions, motivational factors, and behaviors associated with the use of handwashing in the case of H1N1 influenza transmission in Korea, Park et al. [22] suggested that public education campaigns about hand hygiene are effective in changing individual hygiene habits during peak influenza transmission periods. In addition, Duan et al. [15] demonstrated that, in the case of COVID-19, a longer education period is associated with more engagement in government-recommended behavior. However, Cvetković et al. [2] empirically showed that education level does not affect preventive measures.

**Hypothesis 7** **(H7).** *Individuals with higher levels of education take more preventive actions against COVID-19.*

#### 2.4.3. Knowledge

Knowledge performs a similar function as education in Hypothesis 7. Functionally, knowledge helps in understanding the structure of risk perception. Leung et al.’s [6] research on the SARS outbreak in Hong Kong showed that respondents were highly knowledgeable about SARS; about four out of five respondents knew that SARS was transmitted by person-to-person droplet nuclei. In addition, 62.3% knew that fomites, or contact with contaminated objects, were also possible media for transmission.

If people have specific knowledge about the risks or causes of COVID-19, preventive actions can be taken frequently. Jose et al. [8] showed that 99.3% of respondents were aware of COVID-19, and 88% had good knowledge. They found that knowledge was significantly related to behavioral change. Kwok et al. [22] showed that a higher perceived understanding of COVID-19 is associated with a higher degree of social distancing practices. Those with high knowledge of COVID-19 followed recommendations suggested by the authorities to avoid infection (88%), engaged in good practices (93.8%), and were more likely to take a vaccine (81.8%).

**Hypothesis 8** **(H8).** *The more knowledge one has, the more COVID-19 preventive behaviors one exhibits.*

#### 2.4.4. Personal Health Status

Being healthy is not only a basic physical need to overcome COVID-19 but also, from a psychological perspective, a means to overcome the resulting anxiety and depression. Better health status, according to physical and subjective evaluations, reduces perceived risk from COVID-19, leading to fewer preventive behaviors. According to Lee and You [9], subjective health is negatively correlated with perceived susceptibility and perceived severity. Leung et al. [6] showed that, in the case of the 2003 SARS outbreak, respondents who had respiratory or febrile symptoms, especially persistent high fever and cough or difficulty breathing, were more likely to adopt precautionary measures to a greater extent. Park et al. [22] also showed that handwashing is more common among people with recent flu-like symptoms.

**Hypothesis 9** **(H9).** *Individuals with better health are less likely to engage in preventive behavior for COVID-19.*

#### 2.4.5. Social Support 

In an emergency, social networks can serve as channels through which people can receive help in response to illness and share related information. In addition, social support or normative pressure from human networks plays a motivating role in coping with disease. Dryhurst et al.’s [41] study showed that social networks have a decisive influence on COVID-19 risk perception and related information obtained from friends and family plays a role as a social extension. The information that people receive from acquaintances affects their perception of COVID-19 risk. These results imply that social support affects preventive actions. Additionally, Lee and You [17] found that social support is negatively correlated with perceived susceptibility and perceived severity. Moreover, Goodwin et al. [46] showed that group discussions with family and friends may reinforce existing anxiety.

**Hypothesis 10** **(H10).** *Greater social support is positively associated with preventive behavior in response to COVID-19.*

## 3. Sample and Measures

This study analyzes survey data (*n* = 1525) collected from Korea’s general population between 6 August 2020 and 11 August 2020. The data were collected from a panel of respondents fielded by a professional public opinion research organization, Hankook Research, and the web survey method was adopted. Each respondent provided informed consent before participating in the survey. Privacy was guaranteed because personal information, such as sensitive IDs and names, were not collected, and individuals cannot be identified based on the collected data. The key sampling issue is its representativeness to the population. To increase the representativeness of the sample, the distributions of the general public in Korea by gender, age, and region were reflected in the sample quota ratios when the data were collected. As a result, the proportions of responses by gender, age, and region in the sample are consistent with those of the population. 

The questionnaire was designed based on previous studies on SARS [6] and COVID-19 [8,15,17,37]. To increase the validity of the questions, five experts reviewed and revised them after conducting a cognitive test. The most important questions were those measuring the preventive behaviors recommended by the government, the WHO, and other scientific organizations. Individual preventive behavioral responses to the threat of the COVID-19 pandemic were measured with 19 items: for example, wearing a mask, covering one’s mouth with one’s sleeve when coughing, and washing hands for at least 30 s. The answers were rated on a five-point scale (1 = do not comply at all, 2 = slightly do not comply, 3 = moderately comply, 4 = somewhat comply, 5 = highly comply). The total value of preventive behavior was calculated by averaging the scores of each respondent for each individual question.

For the other independent variables, respondents were asked to give their opinions on specific statements. Responses were measured on a five-point scale. For concepts that were evaluated using two statements, a reliability test was executed. The results are shown in Table 2, which also indicates that the Cronbach’s alpha for all measures met the standard of greater than 0.60.

Some items do not use a five-point scale. Four types of question forms were used to measure the variables. First, respondents were asked, “Did anyone you know have a confirmed case of coronavirus? (1) No (2) Yes.” Second, the statements about one’s family health status were as follows: “There is a person in the family who catches a cold easily” (statement 1) and “We usually catch a cold easily” (statement 2), with five possible options (1 = I do not agree at all, 2 = I disagree, 3 = It is neutral, 4 = I agree, 5 = I completely agree). Third, to measure exposure to media, the respondents were asked how much COVID-19-related information they obtained from the following sources: (1) offline media (broadcasting, paper newspapers, magazines, etc.), (2) online media (Internet newspapers, portal news, etc.), and (3) Internet sources (personal blogs, social networks, cafes, and communities). Responses to this were on a five-point scale: (1) I did not get information at all, (2) I did not get much information, (3) I got information, (4) I got some information, and (5) I got a lot of information. In the case of income, 11 categories were defined, and respondents could select any one option. Fourth, in the case of education, seven categories, with 1 point for no formal education to 7 points for a doctoral degree or higher, were defined. 

## 4. Analysis and Findings

### 4.1. Descriptive Analysis

**Figure 1:**
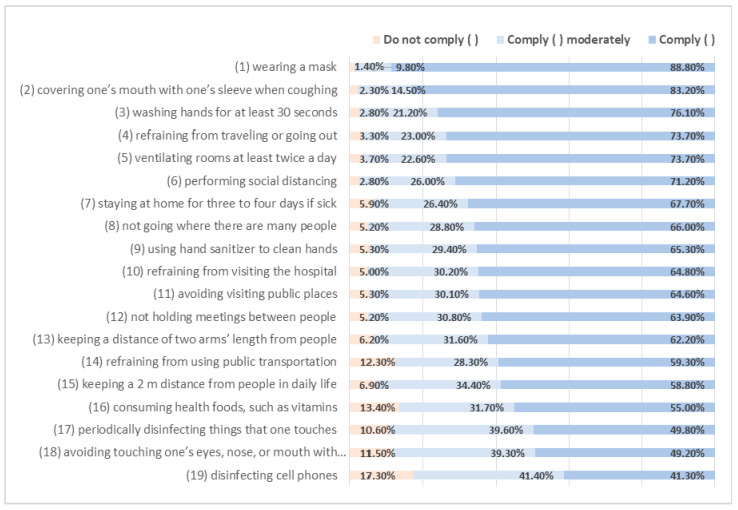
Frequency of preventive behaviors.

First, Figure 1 shows a basic frequency analysis of 19 preventive behaviors for COVID-19. For the analysis, items that were originally measured on a five-point scale were divided into three groups: Group 1 (“do not comply”) includes all respondents who said “I do not comply at all” or “Slightly do not comply,” Group 2 (“do moderately”) includes all those who responded “I moderately comply,” and Group 3 (“comply”) includes all who responded “I somewhat comply” or “I comply too much”. The survey found that 98.6% of the respondents wore face masks. This high rate contrasts with previous findings for other pandemic diseases. During the 2003 SARS outbreak in Hong Kong, 75.8% of people wore a face mask, and 59.9% of people wore a face mask during the pandemic influenza event in Australia in 2008 [12]. In this study, the results for other preventive behaviors showed that more than 80% of the respondents engage in these behaviors. This finding is interpreted as resulting from the high level of fear of COVID-19 based on the higher possibility of infection. Among the preventive actions, several items show a high degree of compliance, and these items are related to government recommendations. The four preventive actions with relatively low levels of compliance are “disinfecting cell phones”; “avoiding touching one’s eyes, nose, or mouth with one’s hands”; “periodically disinfecting things that one touches”; “consuming health foods, such as vitamins.” The commonalities between these items are that they are not official government recommendations, they require considerable effort in practice, and they are not easy to implement unless they are solidified by behavioral habits.

**Figure 2:**
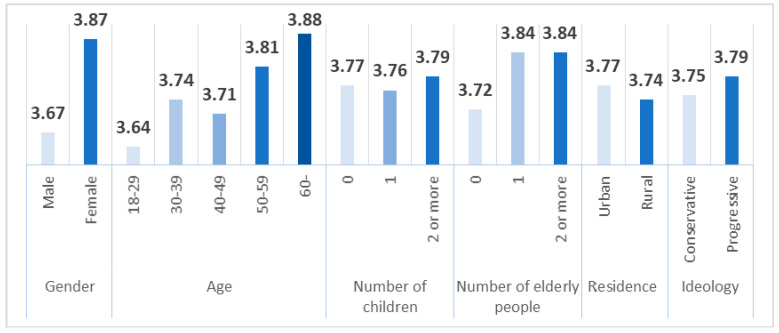
Means by socio-demographic group.

Next, to see how COVID-19 response behaviors vary across demographics, the average scores presented in Figure 2 are based on all 19 behaviors. We first derived the average of these 19 scores of preventive behaviors. Next, we calculated the average value of compliant behavior by gender, age, number of children and elderly people in one’s family, residential area, and ideological group. The results are shown in Figure 2.

Women engage in more recommended behaviors than men do, and this difference is statistically significant (F = 52.708, *p*-value = 0.000). This may be because women are more sensitive to risk than men. Similar to our findings, Prati et al. [39] showed that women engage in more recommended behaviors to prevent epidemic diseases than men do. Moreover, women adopted recommended behaviors more than men did in Hong Kong [5] and Serbia [2] during the COVID-19 epidemic and in Korea [22] during the influenza epidemic of 2010. Lau et al. [11] showed that, in the case of the 2009 H1N1 epidemic in Hong Kong, women regularly wore masks and washed their hands more than ten times a day more often than men did.

Protective behavior also increases with age. The difference between age groups was statistically significant in our samples (F = 9.119, *p*-value = 0.000). As people age, their health weakens, and it is natural that older people’s chances of defensively pursuing health-related behaviors are greater. However, among the five age groups, people in their 40s exhibited less preventive behavior than did people in their 30s. In previous studies, Kwok et al. [22] showed that social distancing activities are high among those in their 50s or older. However, Cvetković et al. [2] demonstrated that age is the most important predictor of preventive measures; younger respondents reported the highest level of restricted movement. They explain that this finding may result from younger people’s higher reception of messages from media and experts and the perception of greater penalties imposed. 

The level of preventive behavior among respondents with two or more children was greater than that among respondents with no children or one child, but this difference was not statistically significant (F = 0.169, *p*-value = 0.845). Those with elderly family members had higher levels of preventive behavior than those without. These results indicate that as the number of children and elderly people in a family increases, compliance with recommended behaviors for COVID-19 prevention increases. Thus, as people associate more with those who are relatively vulnerable to COVID-19, they become more likely to act to prevent it. Similarly, Ibuka et al. [47] showed that respondents from larger households express stronger interest in taking medications and engage in more precautionary activities. However, the group differences in our data were not statistically significant (F = 0.561, *p* = 0.454).

The degree of compliant behavior is higher in urban areas than in rural areas. These results were not statistically significant (F-value = 1.464, *p* = 0.226). However, Ibuka et al. [39] confirmed that precautionary behaviors in response to H1N1 influenza differed, not only temporally but also geographically. According to Kwok et al. [5], the adoption of social distancing was high among people living in the New Territories, east of Hong Kong. Dual et al. [5] showed that urban dwellers engage in more government-recommended behaviors than do rural dwellers.

Ideologically, the level of precautionary behaviors is greater among progressive respondents than among conservative ones. In the case of Korea, conservative Christianity has developed conspiracy theories about COVID-19, and this tendency is interpreted as leading to low preventive actions by conservatives. However, this difference was not statistically significant (F = 1.44, *p* = 0.226). 

### 4.2. Regression Analysis

To identify the predictors of preventive actions, we conducted a regression analysis by setting sociodemographic, health belief, and resource factors as explanatory variables. Dependent variable is a simple calculated means of fifteen preventive actions. The results are presented in Table 3. Models 1 to 3 show the determinant structures for each of the three factors, and Model 4 shows the full model.

Model 1 shows how sociodemographic variables influence preventive measures for COVID-19. The six demographic variables explain only 6.1% of the variance in preventive actions. Among them, gender (female), age, number of elderly people and children in one’s family, and ideology (progressive) positively influence preventive measures. First, women respond more actively than men do. According to Ibuka et al. [47], women have a higher perceived likelihood of influenza infection, a greater willingness to take drugs, and more involvement in information-seeking activities than men. In addition, Cvetković et al. [2] found gender differences in the adoption of preventive measures. This gap could be explained by women’s traditional roles in Serbia, with women concentrating more on family or household care. Such gender role divisions are more sensitive to pandemic issues. Second, as age increases, people take more preventive actions, potentially because the elderly have weaker health. Cvetković et al. [2] showed that age is positively related to preventive behavior. According to Leung et al. [6], who studied the SARS pandemic, as age increases, the likelihood of engaging in proactive defensive behavior increases. Third, as the number of elderly people or children in one’s family increases, preventive behaviors for COVID-19 increase. This result is interpreted as people engaging in more safety practices when family members are vulnerable to COVID-19. More progressive ideologies are associated with more preventive actions. This finding contradicts Dryhurst et al. [41], who found that political ideology influences COVID-19 risk perception, which, interestingly, differs from country to country. In the United Kingdom and the United States, conservatives have low perceptions of COVID-19 risk, but in Korea and Mexico, perceptions are high. Finally, residential area does not affect the adoption of protective actions, which differs from Chen and Chen’s [14] findings that, compared to urban residents, rural residents are less likely to engage in preventive behaviors and more likely to hold negative attitudes about the effectiveness of engaging in preventive behaviors. 

Among the five sociodemographic variables, gender had the largest standardized coefficient, followed by age. This result is natural because both women and the elderly are typically vulnerable to crises.

Model 2 shows that among the eight independent variables constituting health belief factors, six were significant. Perceived severity, perceived barriers, perceived benefit, self-efficacy, poor family health, and media exposure had positive effects on COVID-19 preventive behaviors, whereas only susceptibility had a negative effect. First, the impact of perceived severity on preventive actions confirms previous research findings. According to Barr [12], people with higher levels of threat perception in Australia were significantly more likely to be willing to comply with specific public health behaviors. Park et al. [22] showed that the frequency of handwashing increases when perceived severity (i.e., “If you were infected with H1N1 influenza, how great of a burden would it be on your daily life?”) increases. In Prati et al.’s [39] study of the 2009 H1N1 influenza pandemic, perceived severity indirectly affected recommended behavior through people’s affective response. In our study, perceived severity directly affected recommended behaviors. Second, the negative influence of perceived susceptibility is contrary to previous findings. As previously mentioned, this result suggests that there may be structural factors that prevent people with a high probability of contracting COVID-19 from actively responding to the threat. Park et al. [22] showed that when perceived susceptibility (i.e., “How possible do you believe it is for you to become infected with H1N1 influenza?”) is high, the frequency of handwashing increases. Furthermore, Prati et al. [39] showed that the likelihood of infection indirectly affects recommended behaviors. Moreover, Leung et al.’s [6] study of the SARS outbreak showed that a higher self-perceived likelihood of contracting SARS was a significant, positive predictor of precautionary measures. Third, it is common for perceived barriers and benefits to exert contrasting impacts. However, in this study, these variables both had a positive relationship with preventive actions. Contrary to our hypothesis, perceived barriers promote preventive actions. This result indicates that the more perceived barriers exist, the more preventive practices are promoted. This is due to the very high risk of COVID-19 and the very high level of response action despite perceived barriers. Fourth, the self-efficacy of preventive actions positively affects preventive responses. After separating individual and collective efficacy, Dryhurst et al. [41] confirmed that both have the same effect on COVID-19 risk perceptions. Interestingly, they had different effects in our study; the former had a positive relationship with risk perception, whereas the latter had a negative one. Fifth, the variables related to cues of action that had significant effects were the health of one’s family and media exposure. When the health of one’s family is poor and exposure to the media increases, compliant behavior in response to COVID-19 increases. Family members and personal ties influence people’s infectious disease-related actions in various ways. For example, family composition and friends’ concerns influence individuals’ behavioral choices. Prati et al. [39] demonstrated that worry from family members and friends indirectly affects recommended behaviors. 

Based on standardized beta values, the self-efficacy of protective behavior has the greatest explanatory power among the eight variables that make up the HBM, followed by perceived severity and media exposure. These results imply that not only the risk factors (i.e., perceived severity) of COVID-19 but also confidence in one’s actions (i.e., self-efficacy) and triggers for action (i.e., exposure to media) lead to protective actions.

Model 3 shows the influence of the five resource factors on preventive behavior. Income and education do not have statistically significant effects. In Cvetković et al. [2], monthly income did not affect preventive measures. However, they show that the content of well-observed behaviors varies by education level; those with a high school degree are the most likely to make isolation plans for their households, whereas those with a masters/doctoral degree have the highest scores for avoiding shaking hands, maintaining the recommended distance, and having food supplies for several months or more. Knowledge, personal health status, and social support positively impacted COVID-19 preventive behaviors in this study. Kwok et al. [5] demonstrated that social distancing increases when people have better knowledge of COVID-19. Knowledge plays a role in amplifying risk perception. In Model 3, social support had the greatest explanatory power, followed by knowledge. These findings suggest that preventive actions against COVID-19 are not influenced solely by health factors. 

Model 4 includes all predictor variables. The differences between Models 1, 2, and 3 are that the residential area and ideological variables no longer have significant effects. Considering the variables in the overall model, self-efficacy (+) has the greatest explanatory power based on the standardized regression coefficient value, followed by gender (female), knowledge (+), personal health status (+), perceived severity (+), social support (+), and age (+). The fact that two of these variables are health belief factors, three are resource factors, and two are demographic factors suggest that preventive behavior is affected by various factors rather than a single one. 

Finally, we consider the explanatory power of Models 1, 2, and 3 based on their R-squared values. Model 2 (health belief factors) has an R-squared value of 17.0%, Model 3 (resource factors) has an R-squared value of 12.8%, and Model 1 (demographic factors) has little explanatory power, 5.7%. These results suggest the importance of health belief factors in explaining preventive actions.

### 4.3. Moderating Analysis

Because this study assumes that the structural properties of resources determine individuals’ beliefs, we analyze how resource factors moderate the relationship between health belief factors and COVID-19-compliant behavior. We do not present a detailed hypothesis on how resource factors moderate the relationship because there were too few previous studies that have built hypotheses and verified the moderating role of resource factors. 

**Figure 3:**
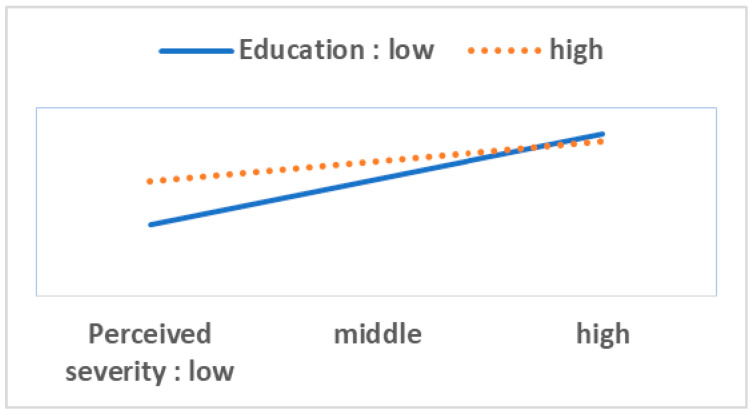
Perceived severity (IV) × Education (M) = Preventive behavior (DV); Note: IV (independent variable), M (moderator), DV (dependent variable).

**Figure 4:**
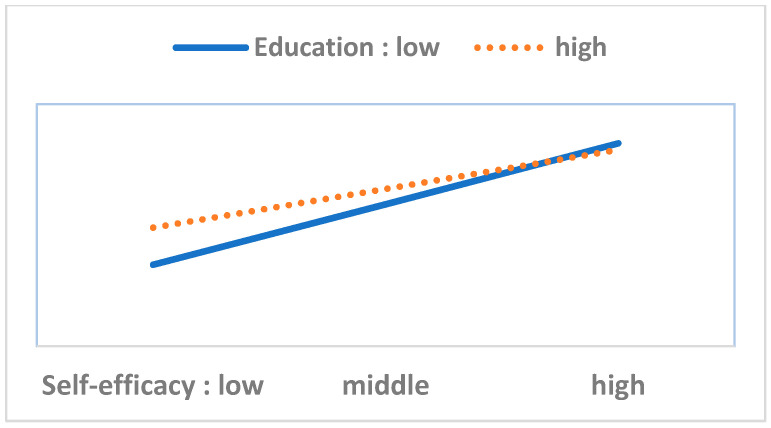
Self-efficacy (IV) × Education (M) = Preventive behavior (DV).

**Figure 5:**
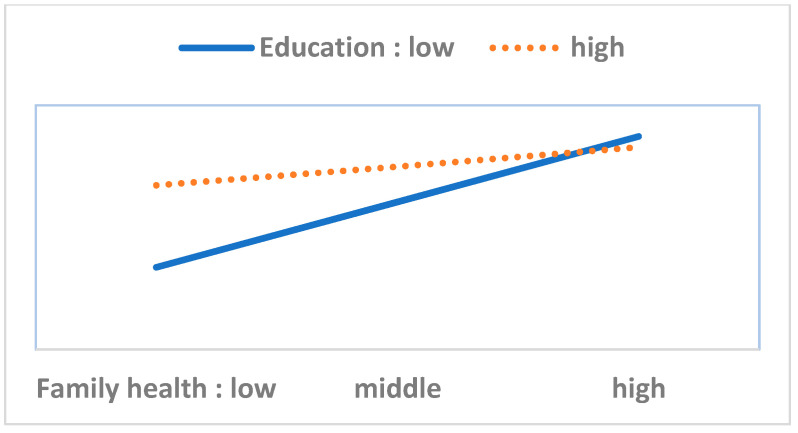
Family health (IV) × Education (M) = Preventive behavior (DV).

**Figure 6:**
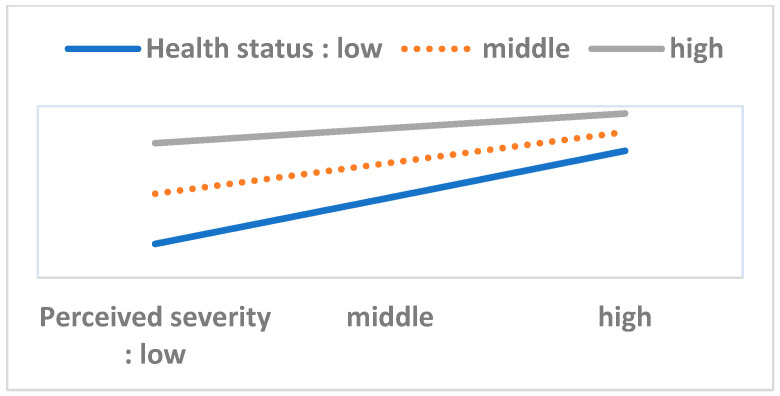
Perceived severity (IV) × Personal health status (M) = Preventive behavior (DV).

**Figure 7:**
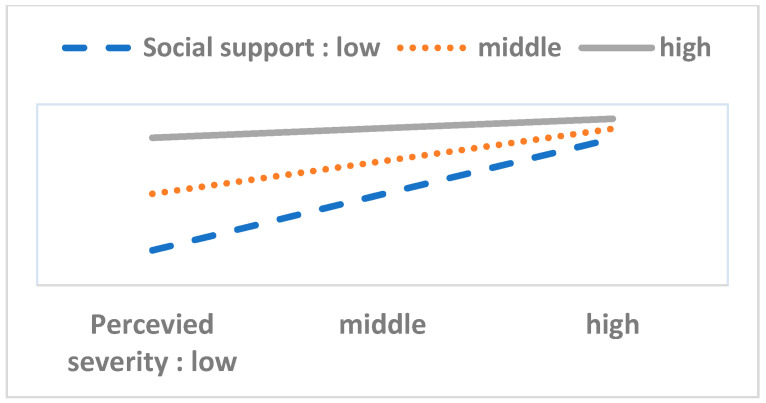
Perceived severity (IV) × Social support (M) = Preventive behavior (DV).

**Figure 8:**
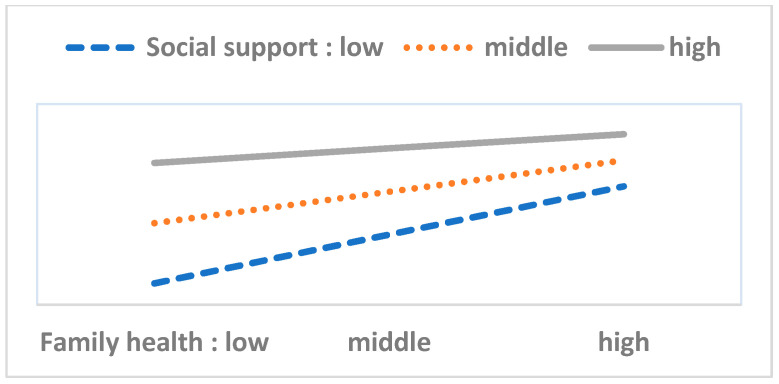
Family health (IV) × Social support (M) = Preventive behavior (DV).

To perform the moderation analysis, we first checked the statistical significance of 40 interaction terms (eight variables in the HBM multiplied by five resource factor variables). Out of these, we found only six significant cases. Next, we performed a simple slope test and drew Figure 3, Figure 4, Figure 5, Figure 6, Figure 7 and Figure 8. The overall interaction analysis follows the procedure proposed by Baron and Kenney [48]. 

Figure 3, Figure 4 and Figure 5 show the moderating effect of education. In Figure 3, perceived severity increases preventive action, but its effect depends on the education level. When perceived severity is low, a high level of education induces preventive action, but when it is high, the effect of education is weakened. In Figure 4, when self-efficacy is high, preventive action is also high, but this effect depends on education. In particular, when self-efficacy is low, the educational effect is remarkable. In Figure 5, weak family health is associated with more preventive behavior, but this effect also depends on the level of education. In other words, when family health is poor, a high level of education leads to more preventive action. Figure 6 shows that when perceived severity increases, preventive action increases. However, this effect varies depending on the individual’s health status, and perceived severity is effective in inducing preventive action when the individual’s health level is good overall. Figure 7 and Figure 8 show the moderating effect of social support. In Figure 7, preventive behavior increases when perceived severity increases, but the effect is higher when social support is strong. 

In this analysis, education level, personal health status, and social support play moderating roles in preventive behaviors. These moderating variables intervene in the effects of perceived severity, self-efficacy, and family health on preventive action. 

## 5. Discussion and Implications

The purpose of this paper is to identify how health belief factors and resource factor theory influence individuals’ preventive actions in response to COVID-19. Also, we examine the moderating role of resource factors in the relationship between predictors and preventive actions. The implications of this study are as follows. First, public responses to various preventive action measures differ in scale. For example, 98.6% of respondents wear masks, indicating that many people engage in this behavior. However, compliance with “disinfecting cell phones”; “avoiding touching one’s eyes, nose, or mouth with one’s hands”; “periodically disinfecting things that one touches”; “consuming health foods, such as vitamins” is relatively low. The commonalities between these items are that they are not official government recommendations; they require considerable effort in practice, and they are not easy to implement unless they are solidified by behavioral habits. These results suggest that it is best to formulate and promote strategies that consider people’s behavioral habits and efforts to engage in protective behaviors.

Second, self-efficacy has the greatest influence on preventive behavior, and gender (female), knowledge, personal health status, perceived severity, social support, and age are also influencing factors. These results imply that strategies should be adopted that increase individuals’ confidence in their ability to take preventive actions and should improve preventive actions without excluding women and the elderly. In addition, programs should focus on strengthening social support through increased interactions within the community. 

Third, to improve citizens’ ability to cope with COVID-19, along with health belief factors, resource factors and demographic variables must also be considered. Knowledge, personal health status, and social support are needed to help convert health beliefs into practice. In terms of resource factors, public relations efforts are needed to spread knowledge about COVID-19; programs for health promotion within the local community must be developed and a human network of support in the community must be established. 

Fourth, our study confirms the moderating effects of education level, personal health status, and social support. This suggests that the role of these three moderating variables should be considered when implementing policy. Particularly, when the levels of perceived severity, self-efficacy, and family health are low, the three moderating variables should be considered. For example, when perceived severity is low, policy measures should strengthen social support. 

## 6. Limitations and Future Research

Our study has some limitations that can be addressed in future work. First, because the determinant analysis was performed at the individual level, additional analyses are needed at the community and national levels. Goodwin et al. [38] show that the national context of a pandemic disease affects response behaviors. Second, this study analyzed the determinants of behavioral actions based on the HBM and resource theory. However, in analyzing the impacts of proactive behaviors, the psychometric paradigm proposed by Paul Slovic [49,50], which stresses subjective perception; the theory of planned behavior, TPB, which emphasizes the intentionality of behavior [51]; a risk communication model that emphasizes the social spread of risk [52] can also be used as a model for comparative analysis. In the future, a comparative analysis of the relative explanatory powers of these models is needed. Third, there is some debate over applying multiple regression analysis when using the Likert scale, wherein R^2^ decreases. However, it was found that such errors decrease when the scale is greater than four points [53]. Moreover, Lubke and Muthen [54] found that it is possible to find true parameter values using Likert scale data, if assumptions about skewness, number of categories, etc., were satisfied. Finally, since we only focused on health beliefs and resource factors, various values, culture, structural context, resources, and communication factors have not been included [55,56,57,58,59,60,61,62,63,64,65,66,67,68,69,70].

## 7. Conclusions

The purpose of this study was to analyze the effects of variables from the HBM and resource theory on COVID-19-related preventive behavior. We summarize our findings as follows. 

First, not all preventive behaviors appear to be similar; wearing a mask is the most frequent preventive behavior. Seond, a simple means analysis showed that women engage in recommended behavior more frequently than men do. This result is believed to be because women are more sensitive to risk. Preventive behavior also increases with age. As people age, their health weakens. As a result, it can be presumed that at-risk groups, such as women and elderly people, are more active in preventive behavior. Third, the results of the regression analysis show that gender (female), age, the number of elderly people in one’s family, perceived severity, perceived benefit, self-efficacy, good family health, media exposure, knowledge, personal health status, and social support positively affect preventive actions. Conversely, perceived susceptibility has a negative effect. Two of the variables have different effects from their hypothesized directions: perceived susceptibility and personal health status. When looking at the explanatory power of variables based on standardized regression coefficient values, self-efficacy has the greatest explanatory power followed by gender (female), knowledge, personal health status, perceived severity, social support, and age. Of the 10 hypotheses, 4 were supported as established in this study, 2 were partially supported, 2 were not significant, and 2 were counter to the hypotheses. Fourth, three resource factors played a moderating role in inducing the effects of HBM variables on preventive behavior. Education level, personal health, and social support served as moderators in the relationship of perceived severity, self-efficacy, and family health with preventive action.

## Figures and Tables

**Table 1:** Health belief model and resource theory.

	Health Belief Model	Resource Theory
Theoretical background	Psychology	Politics, sociology, economics
Main variables	Perceived susceptibility/severity, perceived benefits/barriers, efficacy, action cues	Economic wealth/income, education, knowledge, social support/networks
Individual behavior	Independent of others	Depends on structural resource constraints
Strengths	Highlights the internal psychological structure of decisions	Shows the external and objective determinants of behavior
Limits	Disregards the context Provides weak general explanations	Dismisses individual perception and cognition

**Table 2:** Measurement and reliability.

Concept	Measures	Reliability
1. Susceptibility	I am more likely to be at risk for COVID-19 than others. I live in an environment where I can be exposed to the COVID-19 infection.	0.759
2. Severity	Diseases caused by the COVID-19 infection have very serious consequences. Diseases caused by the COVID-19 infection will have a major impact on my life.	0.781
3. Perceived barriers	Excessive efforts are being made to comply with actions for COVID-19 prevention. There are many obstacles to complying with actions for COVID-19 prevention.	0.503
4. Perceived benefit	The benefits outweigh the costs of complying with actions for COVID-19 prevention. The benefits outweigh the inconvenience of following actions for COVID-19 prevention.	0.575
5. Self-efficacy	If I try, I can fully practice preventive actions. I have enough ability to take actions for COVID-19 prevention.	0.865
6. Knowledge	I am familiar with the COVID-19 infection. I know more about COVID-19 than others.	0.840
7. Personal health status	I am healthy. I am in good health compared to other people.	0.901
8. Social support	I have good relationships with a lot of people. I can get help from others when I’m in trouble.	0.800

**Table 3:** Multiple regression analysis.

			Model 1			Model 2			Model 3			Model 4		
			B	SE	Beta	B	SE	Beta	B	SE	Beta	B	SE	Beta
Constant			3.182	0.067		2.106	0.147		2.507	0.086		1.071	0.156	
Sociodemographic factors	Gender (female)		0.197 ***	0.027	0.181							0.184 ***	0.024	0.169
	Age		0.005 ***	0.001	0.125							0.004 ***	0.001	0.105
	Number of elderly people		0.078 **	0.033	0.071							0.097 ***	0.03	0.088
	Number of children		0.057 *	0.032	0.047							0.046	0.028	0.038
	Residence (rural)		−0.026	0.041	−0.016							−0.002	0.036	−0.001
	Ideology (progressive)		0.049 *	0.028	0.045							−0.034	0.025	−0.031
Health belief factors	Perceived susceptibility					−0.041 **	0.017	−0.062				−0.038 **	0.016	−0.057
	Perceived severity					0.104 ***	0.017	0.151				0.085 ***	0.017	0.124
	Perceived barriers					−0.009	0.018	−0.012				−0.015	0.017	−0.02
	Perceived benefit					0.057 ***	0.019	0.075				0.032 *	0.018	0.043
	Self-efficacy of preventive behavior					0.22 ***	0.02	0.29				0.171 ***	0.019	0.225
	Action cues	Knowing someone who is infected				−0.02	0.075	−0.006				−0.027	0.07	−0.009
		Family health (poor)				0.031 *	0.016	0.046				0.051 ***	0.015	0.077
		Media exposure				0.083 ***	0.017	0.118				0.047 ***	0.016	0.066
Resource factors	Income								−0.021	0.03	−0.017	−0.007	0.027	−0.005
	Education								−0.03	0.027	−0.027	0.038	0.026	0.034
	Knowledge								0.149 ***	0.021	0.177	0.112 ***	0.02	0.133
	Personal health status								0.102 ***	0.017	0.15	0.089 ***	0.017	0.132
	Social support								0.146 ***	0.021	0.184	0.094 ***	0.019	0.118
F-value			16.328 ***			40.710 ***			44.518 *			31.639 ***		
R^2^/Adjusted R^2^			0.061/0.057			0.177/0.173			0.128/0.125			0.286/0.277		

Note: * *p* < 0.05; ** *p* < 0.01; *** *p* < 0.001.
